# Single-Input Multi-Output U-Net for Automated 2D Foetal Brain Segmentation of MR Images

**DOI:** 10.3390/jimaging7100200

**Published:** 2021-10-01

**Authors:** Andrik Rampun, Deborah Jarvis, Paul D. Griffiths, Reyer Zwiggelaar, Bryan W. Scotney, Paul A. Armitage

**Affiliations:** 1 Academic Unit of Radiology, Department of Infection, Immunity & Cardiovascular Disease, University of Sheffield, Sheffield S10 2RX, UK; y.rampun@sheffield.ac.uk (A.R.); deborah.jarvis@sheffield.ac.uk (D.J.); p.griffiths@sheffield.ac.uk (P.D.G.); 2 Department of Computer Science, Aberystwyth University, Wales SY23 3DB, UK; rrz@aber.ac.uk; 3 School of Computing, Ulster University, Jordanstown, County Antrim BT37 0QB, Northern Ireland, UK; bw.scotney@ulster.ac.uk

**Keywords:** foetal brain segmentation, MRI, U-Net, HED network, deep learning, convolutional neural network

## Abstract

In this work, we develop the Single-Input Multi-Output U-Net (SIMOU-Net), a hybrid network for foetal brain segmentation inspired by the original U-Net fused with the holistically nested edge detection (HED) network. The SIMOU-Net is similar to the original U-Net but it has a deeper architecture and takes account of the features extracted from each side output. It acts similar to an ensemble neural network, however, instead of averaging the outputs from several independently trained models, which is computationally expensive, our approach combines outputs from a single network to reduce the variance of predications and generalization errors. Experimental results using 200 normal foetal brains consisting of over 11,500 2D images produced Dice and Jaccard coefficients of 94.2 ± 5.9% and 88.7 ± 6.9%, respectively. We further tested the proposed network on 54 abnormal cases (over 3500 images) and achieved Dice and Jaccard coefficients of 91.2 ± 6.8% and 85.7 ± 6.6%, respectively.

## 1. Introduction

The study of brain development in utero is an important research field for early diagnosis and intervention during pregnancy. While ultrasound scanning is well-established for routine foetal screening, magnetic resonance imaging (MRI) is performed clinically in cases where a suspicious abnormality is detected during ultrasound scanning and has been shown to improve diagnostic accuracy and confidence [1]. It is now possible to acquire 3D volumetric in utero MR imaging (iuMRI) of the fetal brain [2]. Automated segmentation of the iuMRI data is highly desirable as it can provide accurate and timely measurements of morphological brain structures (e.g., volume) which can be used for monitoring and characterising fetal brain development. According to a recent review conducted in 2018 by Makropoulos et al. [3], limited studies have attempted to extract the foetal brain using deep learning approaches. This might be due to a lack of annotated foetal brain data. In comparison to adult studies, foetal brain segmentation is a challenging task as the boundary of the foetus is less visible, particularly towards the bottom and top slices, which can lead to under-segmentation. On the other hand, in more central slices, the foetal and skull boundaries might be overlapping, resulting in over-segmentation. Furthermore, in foetal MRI, the brain often occupies a relatively small portion of the imaging field of view (FOV) and its location and orientation within the FOV can be highly variable.

In this study, we propose SIMOU-Net, a hybrid network inspired from the U-Net [4] and fused with the holistically nested edge detection (HED) [5] architectures. Single-Input Multi-Output U-Net (SIMOU-Net) inherits the advantages of both U-Net (combines location information (down-sampling) and contextual information (up-sampling)) and HED (combines multi-scale and multi-level contextual information via a fusion procedure) networks. In this study, multi-scale information refers to the representation of global and local features at different convolutional layers within the network architecture. In contrast, multi-level information refers to the representation of global and local features at different hierarchies within the network architecture. Our motivations are three-fold:

(1) The U-Net architecture combines both location and contextual information but does not combine information collectively (e.g., making a prediction from different prediction maps). As a result, it relies on a single prediction map derived from the previous layers. In machine learning, many studies [6,7,8] have shown that ensemble learning reduces the variance of predictions and reduces generalisation error, often producing better results.
(2) By contrast, the HED network does combine local and global information collectively (e.g., features from different side outputs are fused) but it does not store spatial and contextual information together, which is essential in semantic segmentation. As a result, from our own experience, the HED network is less accurate in cases where the boundary of the foetal brain is less visible.
(3) The majority of the ensemble-based networks in the literature are (i) multi-stream architectures where several independent networks are placed into a single architecture to produce a single prediction map, or (ii) involve separate training of several independent networks to produce different prediction maps, that are averaged to produce a final prediction. SIMOU-Net bypasses the need for training several different networks independently (to combine the predictions).

Our study has the following contributions:

(1) We propose SIMOU-Net, an ensemble deep U-Net inspired from the U-Net and HED architectures which combines multi-scale and multi-level local and global information collectively together with spatial information.
(2) To the best of our knowledge, this is the largest cross-validated foetal brain segmentation study in the literature covering 254 cases (normal and abnormal) with over 15,000 images in total.
(3) To further evaluate the robustness and generalisability of the proposed method, we train our network on normal cases and test it on abnormal cases, which can be extremely challenging due to differences in shape/geometry information. To the best of our knowledge, it is the first study in the literature to have performed this kind of investigation/evaluation.
(4) Most of the fetal brain segmentation studies in the literature evaluate their results on a 2D slice-by-slice basis. From a neuro-radiology point of view, volumetric information is one of the key elements in abnormality detection. Thus, we further evaluate our method by comparing the fetal brain volumes generated automatically with those obtained manually both for normal and abnormal cases.

## 2. Related Work

In foetal brain segmentation, the majority of existing studies are based on conventional image processing (e.g., level set, superpixel and region growing) and machine learning techniques (e.g., Support Vector Machine or ensemble classifiers). In an early study, Anquez et al. [9] developed a method that relies on detecting the foetus’ eyes. In a similar approach, Taleb et al. [10] proposed a method based on age-dependent template/atlas matching. The method defines a region of interest (ROI) using the intersection of all scans and then registers this ROI to an age-dependent foetal brain template. This is followed by refining the initial segmentation to a final segmentation map using a fusion step. The disadvantage of these early methods is the dependency on prior knowledge which can be unreliable. To overcome the requirement for prior knowledge, Alansary et al. [11] proposed a method using the simple linear iterative clustering (SLIC) [12] technique to extract all superpixels in an image followed by feature extraction in each superpixel. Subsequently, superpixels with similar features were merged and the random forest classifier was employed to differentiate brain and non-brain regions. They reported a Dice score of 73.6% based on 55 cases when compared to expert manual segmentations. A semi-automatic method was proposed by Link et al. [13], where a user has to initialise the method by drawing a circle around the brain. This is followed by a region growing technique to estimate the brain region. Their experimental results reported a correlation value of *r*^2^ = 0.91 for the brain volume when compared with manual segmentation based on 199 fetal brains of normal cases. Attallah et al. [14] presented a method that uses an elaborate combination of adaptive thresholding, morphological and watershed operations in order to segment the fetal brain. The data used appears to be single slice data from three central orthogonal planes rather than 3D volumetric data. They report encouraging Dice scores of 0.975 for 50 of the 227 scans, but do not report data from the remaining 177 scans, nor do they report how the ground truth data for comparison was obtained. This work was primarily concerned with the classification of foetal brain abnormalities, with the segmentation being a step in that process. From our own experience, intensity-based methods such as the region growing and SLIC often fail to segment the foetal brain region toward the top and bottom slices due to obscure boundaries and the brain having a similar intensity to surrounding tissues.

To overcome the disadvantages of intensity-based methods, Ison et al. [15] proposed a texture-based method. Firstly, the random forest (RF) classifier with 3D Haar-like descriptors was used to roughly estimate the location of the foetus’ head. The probability map segmentation is refined by taking centroids with high probabilities and optimised using a Markov random field (MRK) appearance model. The method was tested on 43 foetal brains and produced 82% and 89% sensitivity and specificity, respectively. Keraudren et al. [16] proposed a similar pipeline with the following steps but used the scale-invariant feature transform (SIFT) features to represent the brain region and the conditional random field (CRF) was employed to estimate the brain boundary. The authors evaluate the performance of their method on 66 foetal brains and report a Dice score of 93%. In contrast, Kainz et al. [17] developed a method that bypasses the head detection step by directly estimating the fetal brain via an ensemble forests classifier using spherical 3D Gabor features. The resulting probability map was further processed via thresholding and a 2D level-set was employed to perform the segmentation. The authors tested the proposed method on 50 cases and reported a Dice score of 90%.

This summary makes clear that the state-of-the-art in foetal brain extraction methods to date is based on a combination of several traditional image processing techniques such as image registration and level set/graph cuts. Each of these techniques has its own parameters to be set and optimised, which ultimately make foetal brain extraction difficult and semi-automatic or supervised rather than fully automatic. More specifically, we identify the following disadvantages:

(1) The template-based methods rely heavily on the alignment of the query images to atlases or make a strong assumption about the orientation and geometry information [18]. As a result, when the foetal brain is structurally abnormal, this approach often produces inaccurate results due to differences in geometry/shape information.
(2) Most of the methods developed require initial detection of the eyes or head in order to aid localisation of the brain. However, in many images, the eyes are not visible and the skull can be obscured particularly towards the bottom and top of the brain. Furthermore, since this initial step is necessary for subsequent steps, it is essential for the first step to achieve 100% localisation accuracy which is often difficult, or time consuming in the case of manual region placement.
(3) The processing steps that generate initial candidates typically result in many false positives. Therefore, in subsequent steps, it is essential to have a robust post- processing method for false-positive reduction.
(4) The 2D level-set and region growing methods often perform poorly when the brain boundary is unclear or obscured. Unfortunately, this is a frequent occurrence in foetal MRI, especially in the bottom and top slices. While the methods might work successfully on the central slices, in some cases the brain and the skull boundaries can be overlapping, resulting in over-segmentation. Furthermore, these methods do not generally work well with low contrast images.

The recent promising results in deep learning-based image segmentation motivate further development of learning-based, geometry-independent and registration-free foetal brain segmentation [18]. Deep learning (e.g., convolutional neural network (CNN)) is becoming a methodology of choice in brain MRI segmentation [19,20,21]. However, in foetal brain segmentation, the number of studies in the literature is rather small. Recently, Salehi et al. [22] proposed a three-pathway automatic foetal brain extraction based on a modified U-Net [4] using auto-context CNN in which intrinsic local and global image features are learned through 2D patches of different window sizes. The authors reported a Dice score of 95.97% based on 75 normal cases. The main disadvantage is that it requires three separate models that need to be run independently to segment the foetal brain (e.g., from coronal, sagittal and axial views). Furthermore, it is essential to obtain manual annotation for each slice from all planes which is highly time consuming even for an expert annotator. In another study, Rajchl et al. [23] extended the GrabCut [24] method to include machine learning by training a neural network classifier from bounding box annotations (which is called DeepCut). A simple variant of the DeepCut method was proposed and compared with a naïve approach to CNN training under weak supervision. The energy function of the densely connected conditional random field (CRF) was used to iteratively update the training targets to achieve semantic segmentation. The experimental evaluation produced Dice scores of 90.3% and 94.1% for the extended GrabCut and DeepCut methods, respectively. Although the results are promising, experimental results suggest that the CRF parameters are inconsistent and need to be tuned manually to maximise their performance. Furthermore, since the training is interrupted after a fixed number of epochs to update class labels, this process makes training very slow [23]. In a more straightforward approach, Khalili et al. [25] trained their network directly (image-to-image prediction) from the whole image. They proposed a modified U-Net architecture using more robust functions to avoid overfitting and reduce the number of features extracted in each convolutional layer. Based on 12 normal cases, they reported a Dice score of 88%.

Ebner et al. [26] proposed a method based on the P-Net network architecture which consists of three stages: (1) a coarse segmentation for brain localisation, (2) fine segmentation with a multi-scale loss function, and (3) brain reconstruction. They reported an average dice score of 93.4% based on two different datasets. Recently, Lou et al. [27] proposed a multi-stage deep supervision U-Net (DS-U-Net). Their method also contained three stages: (1) a coarse segmentation using the original DS U-Net introduced by Dou et al. [28] to define a 3D bounding box to find the location of the brain, (2) using another DS U-Net [28] to focus on the extracted region to produce finer segmentation and (3) refining the final segmentation by using another DS-U-Net but training it using a local image patch strategy. The authors reported a dice score of 91.68% based on 43 cases. The main disadvantage of these methods [26,27] is that they require three training phases which is very time consuming.

## 3. Materials and Methods

**Figure 1:**
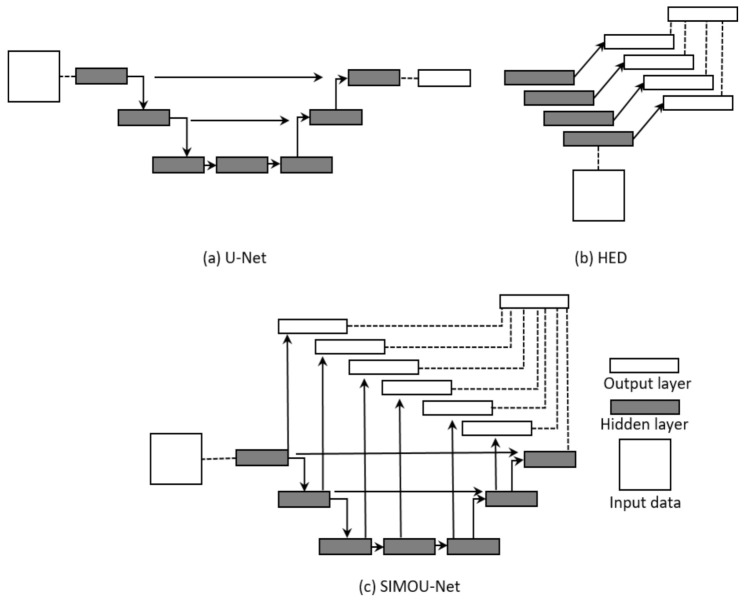
A high-level view of the U-Net, HED and SIMOU-Net architectures.

The SIMOU-Net architecture comprises a single stream deep network with multiple side outputs which promotes an ensemble learning scheme. Figure 1 shows the semantic architecture of the U-Net, HED and SIMOU-Net architectures.

### 3.1. Network Architecture

**Figure 2:**
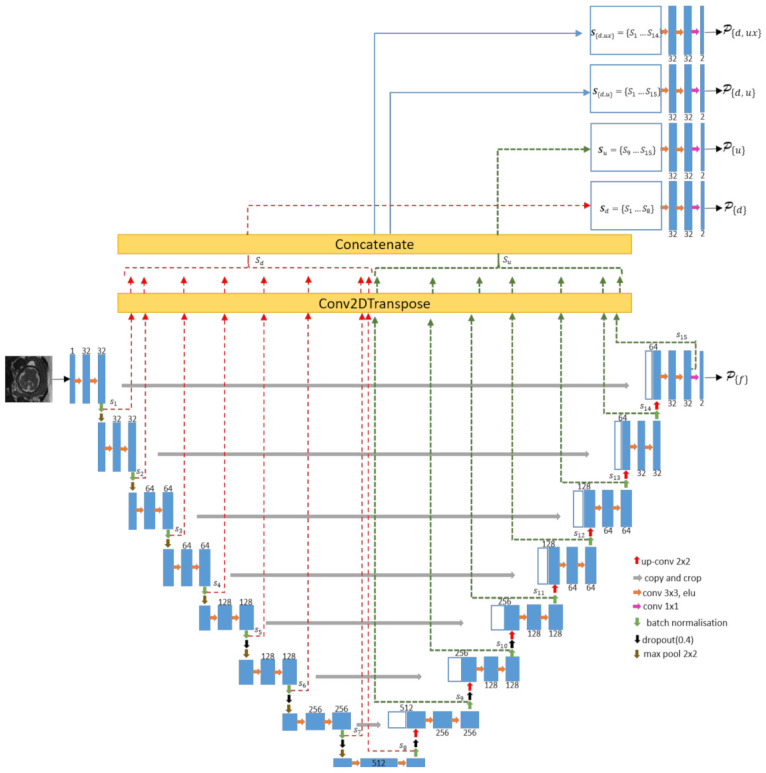
The SIMOU-Net architecture. Prediction maps for each output are denoted as *P*_{}_. Note that in this architecture (and also in the classical U-Net) the downsampling path extracts global information and the upsampling path extracts local information. The ‘Conv2DTranspose’ is also known as deconvolution, which is similar to ‘UpSampling2D’. However, the main difference is that it both performs the upsample operation and interprets the coarse input data to fill in detail while it is upsampling. The concatenation operation merges together the side outputs *s_d_* and *s_u_* using Equation (1), enabling production of the four prediction maps *P*_{*d*}_, *P*_{*u*}_, *P*_{*d,u*}_ and *P*_{*d,ux*}_.

Figure 2 shows details of the SIMOU-Net architecture. The lower part of the network is similar to the original U-Net architecture except it has eight levels of convolutional blocks instead of five. Our deeper architecture enables us to extract finer texture features. The left side of the network consists of repeated application of two 3 × 3 unpadded convolutions followed by an exponential linear unit (‘elu’). Each level of convolutional block is followed by batch normalisation and a 2 × 2 max pooling operation with stride 2 for downsampling.

At each downsampling step, there is one side output (*s_n_*, where *n* = {1, 2,..., 15}, there are 15 side outputs in total) and we use the following number of feature channels: 32, 32, 64, 64, 128, 128, 256, 512. A dropout operation is applied in the last three layers of the downsampling path and the first three layers of the upsampling path. This is to avoid overfitting due to the number of features in this section of the network being very dense. For the right side (expansive path) of the network, each step consists of upsampling of the feature map from the contracting path followed by a 2 × 2 convolution. To optimise the expansive process, instead of using the conventional upsampling operation (e.g., nearest neighbour interpolation) we use the 2D transpose convolution to fill in details, as well as upsampling the image during the model training process. The concatenation operation with the corresponding cropped feature map from the contracting path and two 3 × 3 convolutions are also applied, each followed by an ‘elu’. A similar process occurs in the contracting path, where each upsampling step has one side output. For the upper part of the network, it consists of a 2D transpose convolution for each side output. In total, the SIMOU-Net contains 15 side outputs (*s*_1_,..., *s*_15_); *s_n_* from the left and right paths of the network are concatenated separately using equation 1. Suppose each side output is an *M* (row) × *N* (column) matrix and *i* and *j* represent each element’s location in *s_n_*. We con- catenate all side outputs (see Figure 2 for more details) from downsampling and upsampling paths $\left(S_{\left\{d,u\right\}}=\left\{s_{1},\;\ldots ,s_{15}\right\}\right)$, without *s*_15_ $\left(S_{\left\{d,ux\right\}}=\left\{s_{1},\;\ldots ,s_{14}\right\}\right)$, downsampling path only $\left(S_{\left\{d\right\}}=\left\{s_{1},\;\ldots ,s_{8}\right\}\right)$ and upsampling path only $\left(S_{\left\{u\right\}}=\left\{s_{9},\;\ldots ,s_{15}\right\}\right)$ by adding each *s_n_* in the third dimension. Hence, the resulting concatenated matrix can be represented as

(1) $$S_{c}=\left[\begin{array}{ccc} s_{n}\left(i,j,p\right) & \cdots & s_{n}\left(i,j+N,p\right)\\ s_{n}\left(i+1,j,p\right) & \cdots & \vdots \\ \vdots & \ddots & \vdots \\ s_{n}\left(i+K,j,p\right) & \cdots & s_{n}\left(i+K,j+N,p\right) \end{array}\right],$$

where *c* ∈ {(*d*, *u*), (*d*, *ux*), *d*, *u*}. Note the additional dimension of *p* of **S***_c_* leading to *M* × *N* × *P*. In Keras, this can be implemented using the concatenate operation by setting axis = 3. After concatenation, a couple of 3 × 3 convolution operations with ‘elu’ are applied to each **S***_c_* (e.g., **S**_{*d,u*}_). At the final layer, we use a 1 × 1 convolution to map each 64-component feature vector to two classes (e.g., brain and non-brain) and in total, the SIMOU-Net architecture produces five output prediction maps. Four of them, (*P*_{*d,ux*}_, *P*_{*d,u*}_, *P*_{*u*}_, *P*_{*d*}_) are produced from the upper part of the network and one (*P*_{*f*}_) is produced by the deep U-Net (see Figure 2). The *P*_{*f*}_ map is also known as the main output of deep U-Net, *P*_{*d,u*}_, *P*_{*u*}_ and *P*_{*d*}_ are known as side outputs and *P*_{*d,ux*}_ is known as the combined output of the side outputs and main output.

### 3.2. Training, Validation and Testing

We denote our input training dataset as $D_{tr}=\left\{\left(I_{q},T_{q}\right),q=1,\;\ldots ,\;Q\right\}$, where sample $I=\left\{x_{r}^{\left(q\right)},\;r=1,\;\ldots ,\left|I_{q}\right|\right\}$ denotes the raw input image and $T_{q}=\left\{y_{r}^{\left(q\right)},\;r=1,\;\ldots ,\left|I_{q}\right|\right\}$, $y_{r}^{\left(n\right)}\in \left\{0,1\right\}$, denotes the corresponding ground truth binary map for image *I_q_*. We use the same formulation for our validation data set *D_vd_* = {(*I_q_*, *T_q_*), *q* = 1, …, *Q*}. For simplicity, we denote the collection of all standard network layer parameters as **W**. In the SIMOU-Net architecture, we have five outputs and each output is associated with a classifier in which the corresponding weights are denoted as w = w^(1)^,..., w^(T)^. We consider the following objective function

(2) $$F_{S_{P}}\left(W,w\right)=\sum_{t=1}^{T}\alpha_{t}L_{S_{P}}^{\left(t\right)}\left(W,w^{\left(t\right)}\right),$$

where $L_{S_{P}}$ denotes the image-level loss function for the network’s outputs and α is the weight. Following the study in [5], the loss function is computed over all pixels in a training image $I=\left\{x_{r}^{\left(q\right)},\;r=1,\;\ldots ,\left|I_{q}\right|\right\}$ and its associated ground truth $T_{q}=\left\{y_{r}^{\left(q\right)},r=1,\ldots ,\left|I_{q}\right|\right\}$, $y_{r}^{\left(n\right)}\in \;\left\{0,1\right\}$. Since the brain region often occupies a relatively small portion of the FOV, we employed the loss function proposed by [29], a combination of binary cross-entropy and Dice coefficient, to offset the imbalance of pixels between foreground and background, which is described as:

(3) $$L_{S_{P}}\left(I_{b},T_{b}\right)=-\frac{1}{B}\sum_{b=1}^{B}\left(\frac{1}{2}I_{b}logT_{b}+\frac{2I_{b}T_{b}}{I_{b}+T_{b}}\right),$$

where *I_b_* and *T_b_* are the flatten predicted probabilities and the flatten ground truth of the *b^th^* image, respectively, and *B* indicates the batch size. We train our network with the root mean square propagation (RMSprop) [30] implementation in Keras with a Tensorflow backend. The initial learning rate (*lr*) and gradient moving average decay factor (ρ) are 0.0003 and 0.8, respectively. To minimise training time (e.g., faster to converge) and to have a better estimation of the gradient of the full dataset (hence it converges faster), we favour a maximum batch size (*bs* = 32) that our machine can accommodate. The number of iterations used per epoch (*E*) is based on the number of samples divided by batch size (#Qbs). We monitor the Dice and Jaccard coefficients, set *E* = 1000 and employ the ‘EarlyStopping’ mechanism on the validation set to stop the training automatically when the loss function value does not decrease after 100 epochs.

In our proposed network, with many convolutional layers and different paths within the network, a good initialization of the weights is essential. Otherwise, parts of the network might give excessive activations, while other parts never contribute. For this purpose, we follow the weight initialization implemented by Ronneberger et al. [4] using the Gaussian distribution with a standard deviation of $\sqrt{2/\eta }$ where *η* is the number of incoming nodes of one neuron. This strategy was chosen due to the architecture of our network (alternating convolution and ReLU/ELU layers) and was further suggested in the study of [31].

During testing, given an image *I*, we obtain five prediction maps which are *P*_{*d,ux*}_, *P*_{*d,u*}_, *P*_{*u*}_, *P*_{*d*}_ and *P*_{*f*}_. Subsequently, ensemble outputs can be obtained by further aggregating these prediction maps using the following equation

*P_arg_* = *arg* (*P*_{*d,u*}_, *P*_{*d,ux*}_, *P*_{*u*}_, *P*_{*d*}_, *P*_{*f*}_) (4)

where *arg* indicates the different operations used, namely ‘mean’ (*P*_AVG_), ‘median’ (*P*_MED_) and ‘maximum’ (*P*_MAX_).

### 3.3. Data Description, Pre-Processing and Experimental Setup

More information on the datasets used in this study can be found in [1,2,32]. Our first dataset consisted of MR images of 200 healthy/normal foetuses with gestational age (GA) ranging between 18 and 37 weeks. The second dataset consisted of MR images of 54 abnormal foetuses with GA between 20 and 34 weeks. All studies were performed on a 1.5T whole-body scanner with images acquired using a 3D FIESTA sequence. Slice thickness varies according to gestational age/size of foetal head (range between 2.0 mm and 2.6 mm). Some of the images contain a degree of motion artefacts that are typical for such acquisitions. For each image, the brain was manually segmented by a radiographer with over 10 years’ experience in foetal MRI (who had been trained by an expert neuro-radiologist) using the ‘3D Slicer’ software. The freehand segmentation was performed on axial images with the sagittal and coronal planes used as additional references to improve accuracy. Each case took approximately two to three hours to segment every slice depending on the complexity of the brain and clarity of the image. These manual segmentations were considered to be the ‘gold standard’ for comparison purposes throughout the rest of this study.

To further enrich the training dataset in this study we applied the following data augmentation, where each input image has a corresponding “augmented” image assigned with a: (i) random rotation range of up to 180°, (ii) zooming in and out with a range of 0.1 to 2.0, and (iii) horizontal and vertical flips. Training and testing were performed on a high-performance computing server with Nvidia DGX-1 graphics cards 8x Tesla P100 GPUs (16GB RAM each) (Santa Clara, CA, USA), dual 20-core Intel Xeon E5-2698 v4 2.2 GHz and 512 GB system RAM (Santa Clara, CA, USA), and all models were implemented in Keras with a Tensorflow backend.

## 4. Results and Discussion

### 4.1. Normal Cases

The following metrics are used to quantitatively evaluate the performance of the method: Jaccard (*J*), Dice (*D*), Sensitivity (*Sen*), Hausdorff distance (*HD*) measured in mm, and mean absolute volume differences (*MAVD*) measured in cm^3^. *TP* (cyan), *TN* (black), *FP* (red) and *FN* (yellow) are true positive, true negative, false positive and false negative, respectively used throughout the paper to illustrate segmentation results.

Table 1 shows the average results using the proposed method based on a stratified 3-fold cross validation. We include in the same group (e.g., training set) all slices/images of the same foetus. We compare our results with the original U-Net [4], wide U-Net (wU-Net) [29], nested U-Net with (U-Net++), without deep supervision (U-Net+++) [29], HED [5], SegNet [33], Att-U-Net [34], DS-U-Net [27], P-Net(S) [26] and a modified U-Net version of [18]. All experiments have the same implementation details, such as the number of epochs, function loss, batch size, etc.

Table 1 shows that the SIMOU-Net produced competitive results across the different metrics. Friedman tests indicated significant differences between methods for all metrics; *J* (*p* < 0.001), *D* (*p* < 0.001), *Sen* (*p* < 0.01), *MAVD* (*p* < 0.001) and *HD* (*p* = 0.002). The Nemenyi post hoc test was performed and significant differences with respect to the *P*_AVG_ prediction map score are indicated in Table 1. It significantly outperforms all other methods based on the *Sen* metric and all but the DS-U-Net based on Jaccard and Dice metrics. The *MAVD* and *HD* metrics were less sensitive to the generally small differences observed between methods, with only the wU-Net (*p* < 0.001) and HED (*p* = 0.029) performing significantly worse than the SIMOU-Net *P*_AVG_ prediction map based on *MAVD* and only the HED network performed worse with the *HD* metric (*p* = 0.001). The *P*_{*d*}_ prediction map performed poorly, and this is expected because features are extracted from only the side outputs of the downsampling path (global features) of the network architecture without taking into account local features from the upsampling path. The SIMOU-Net produced the highest *J* = 88.9%, *D* = 94.2%, *Sen* = 97.5% and lowest *MAVD* = 4.2 cm^3^ and *HD* = 7.5 mm when all prediction maps are aggregated using the averaging procedure (*P*_AVG_). Many classification and segmentation deep learning studies [6,7,8] have shown that combining models/prediction maps often leads to better classification results because it reduces the variance of the predictions and generalization error. Using the ‘median’ (*P*_MED_) operation, the SIMOU-Net produced *J* = 87.2%, *D* = 93.3%, *Sen* = 95.9%, *MAVD* = 4.4 cm^3^ and *HD* = 7.6 mm which are close to those from the ‘mean averaging’ operation and only significantly worse for the *Sen* metric. When using the ‘maximum’ operation (*P*_MAX_), we achieved lower accuracy than *P*_AVG_, *P*_MED_, *P*_{*d,ux*}_, *P*_{*d,u*}_, *P*_{*u*}_ and *P*_{*f*}_. This is expected because this operation tends to include outliers in the probability map.

Combining all side outputs (*P*_{*d,ux*}_) produced *J* = 86.9%, *D* = 93.8%, *Sen* = 95.9%, *MAVD* = 4.5 cm^3^ and *HD* = 7.7 mm. Including *S*_15_ (*P*_{*d,u*}_) produced very similar results. These experimental results suggest that there is useful information from the side outputs of the network, as *P_{d,ux}_* and *P_{d,u}_* tended to produce slightly better results than *P*_{*f*}_ alone, although the differences did not reach statistical significance. This might be due to the loss of information because of repetitive convolution operations in the contraction and expansion paths, resulting in the slightly worse result in *P*_{*f*}_.

**Figure 3:**
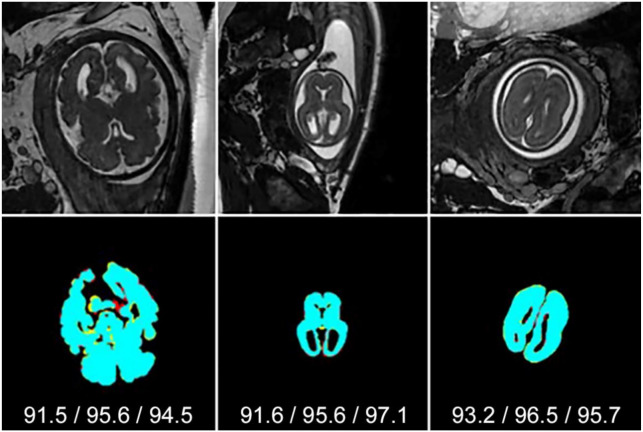
Typical examples of *P*_AVG_ segmentation results from middle slices. Black, cyan, red and yellow represent *TN*, *TP*, *FP* and *FN*, respectively. Jaccard/Dice/Sensitivity scores are shown under each segmentation.

**Figure 4:**
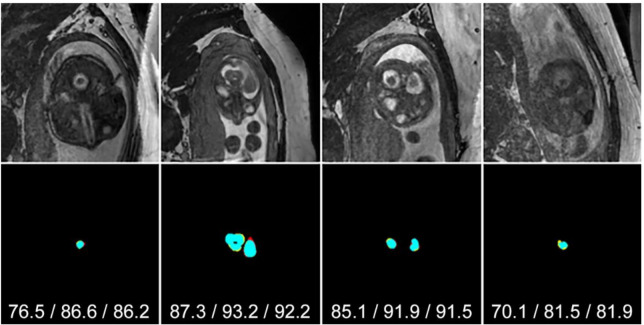
Typical examples of *P*_AVG_ segmentation results from top and bottom slices. Black, cyan, red and yellow represent *TN*, *TP*, *FP* and *FN*, respectively. Jaccard/Dice/Sensitivity scores are shown under each segmentation.

Figure 3 and Figure 4 show examples of segmentation results using *P*_AVG_ for middle and bottom/top slices, respectively. Black, cyan, red and yellow represent *TN*, *TP*, *FP* and *FN*, respectively. In most cases, segmenting the brain from the bottom/top slices is far more difficult than in central slices. This is because the brain region is very small and tends to be more obscure due to low contrast or noise. Figure 3 shows that in all examples the SIMOU-Net produced both *J* > 90% and *D* > 90% whereas for bottom/top slices in Figure 4 the SIMOU-Net produced *J* = 70% to *J* = 90% for very challenging cases.

### 4.2. Abnormal Cases

We further evaluated the proposed network architecture by training it on 180 normal cases, validating it on 20 normal cases (in total 200 normal cases from the first dataset) and then testing the model on 54 abnormal cases. The abnormal cases consisted of the following abnormalities: agenesis of corpus callosum (ACC), mild ventriculomegaly (MV) and structurally deformed. From a radiological perspective, ACC manifests as a partial or wholly absent corpus callosum, MV refers to larger than normal cerebral ventricles and structurally deformed covers a wide range of structural brain abnormalities. From a neuro-radiology point of view, estimating the brain volume from these cases can be difficult due to extreme and obscure brain shape. In fact, it is also difficult for an observer to annotate these cases without substantial experience in dealing with abnormal cases.

Experimental results from the abnormal cases are shown in Table 2. Friedman tests indicated significant differences between methods for *J* (*p* < 0.001), *D* (*p* < 0.001) and *Sen* (*p* < 0.001), but not for *MAVD* (*p* = 0.73), nor *HD* (*p* = 0.79). The Nemenyi post hoc test was performed for the three metrics showing differences and significance with respect to the *P*_AVG_ prediction map score is indicated in Table 2. The SIMOU-Net architecture (*P*_AVG_) achieved *J* = 85.7%, *D* = 91.2%, *Sen* = 93.2%, *MAVD* = 7.1 cm^3^ and *HD* = 8.5mm. When using the ‘median’ operation (*P*_MED_), it produced *J* = 83.7%, *D* = 89.3%, *Sen* = 92.4%, *MAVD* = 7.2 cm^3^ and *HD* = 8.7 mm, which was not significantly different from *P*_AVG_ for any metric. These results presented in Table 2 once again suggest that using the ‘mean’ and ‘median’ operations, or taking account of features from side outputs, tends to produce a better result than relying on a single predictive map, although the differences are small and do not generally reach significance with the exception of *P*_{*d*}_ and *P*_{*u*}_. Some of these differences may begin to reach significance in a larger dataset, as is indicated by the data presented in Table 1 which is based on 200 cases, as opposed to Table 2 which is derived from 54 cases.

In comparison to the other methods, the P-Net(S) and DS-U-Net produced very similar results to our method across different metrics, similar to what was reported in Table 1. The Att-U-Net also showed comparable performance to SIMOU-Net when using the *D* metric. All other networks perform significantly worse than SIMOU-Net when using the *J*, *D* and *Sen* metrics. Similar to Table 1, the *MAVD* and *HD* show less sensitivity to the performance differences between networks with no significant differences found. We expected these results because the DS-U-Net architecture combines its prediction map (e.g., *P*_{*f*}_) with several side outputs from the upsampling path. The Att-U-Net clearly improved the performance of the classical U-Net by at least 2% for the *J*, *D* and *Sen* metrics.

The SegNet produced similar results to U-Net2, U-Net++ and U-Net+++. The HED produced the worst results with *J* = 70.1%, *D* = 75.2% and *Sen* = 79.6%, *MAVD* = 8.2 cm^3^ and *HD* = 11.5 mm. This may be due to the network being less robust at storing spatial information, which is essential in semantic segmentation.

### 4.3. Fetal Brain Volume Estimation

**Figure 5:**
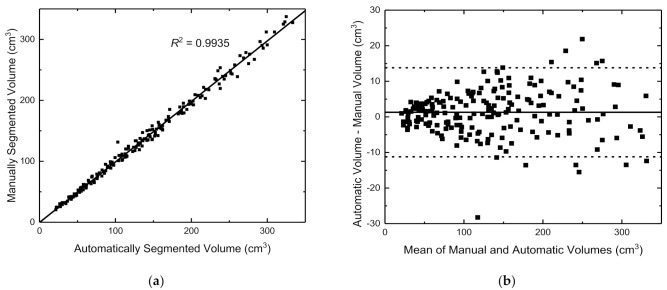

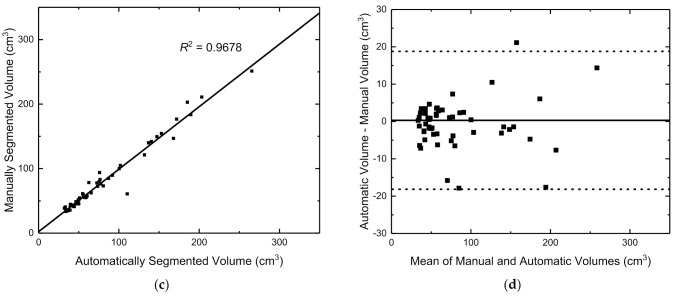
Correlation between manual and automated (*P*_AVG_) foetal brain volume measurements for (**a**) 200 normal cases and (**c**) 54 abnormal cases. Bland–Altman plots showing the limits of agreement between automatic and manual foetal brain volumes for (**b**) the 200 normal cases and (**d**) the 54 abnormal cases.

We also evaluate the robustness of the proposed architecture by comparing the volumes generated automatically with those obtained manually. We compute brain volume by calculating the segmented area for each slice and sum it over the total number of slices, taking account of the pixel dimension and slice thickness. Figure 5a demonstrates that there is an excellent correlation between the automatically generated brain volumes (using the *P*_AVG_ segmentations) and those obtained from the expert manual segmentations (*R*^2^ = 0.9935, *p* < 0.001) for the 200 normal healthy foetuses. Figure 5b shows the corresponding Bland–Altman plot which indicates that there is good overall agreement with a mean difference between the measurements of 1.29 cm^3^ (95% limits of agreement {−11.22 cm^3^, 13.80 cm^3^}), which suggests that the proposed method tends to slightly overestimate the brain volume. Both graphs demonstrate that the distribution of volumes spreads more as the volume increases. This is due to the more complex appearance of the brain in older foetuses (e.g., above 30 weeks) compared to younger foetuses, which makes the brain more challenging to segment due to increased partial volume effects and there may also be more intrinsic inter-subject variation by this age.

Similarly, Figure 5c demonstrates that there is an excellent correlation between automated and manual volume measurements in the 54 abnormal cases (*R*^2^ = 0.9678, *p* < 0.001). The corresponding Bland–Altman plot in Figure 5d once again suggests that the proposed method tends to slightly overestimate brain volumes compared to the expert gold standard (mean difference 0.31 cm^3^, 95% limits of agreement {−18.17 cm^3^, 18.79 cm^3^}). While the mean bias is slightly lower for the abnormal cases, the limits of agreement indicate a greater amount of variability in the measured volumes when compared to the gold standard expert segmentations. This is expected due to (i) the model being independently trained/validated on normal cases only and (ii) that some of the abnormalities introduce a more complex appearance to the brain, which, like the older foetuses, makes the brain harder to segment reliably. Nevertheless, the automated method produced promising results with most estimated volumes lying very close to the straight line. This gives us confidence that the method will provide a reliable segmentation in a wide range of abnormalities, even when the model is trained on normal healthy cases. This is particularly useful for rare pathologies, where it may be difficult to build up large training sets of abnormal cases.

### 4.4. Visual Comparison with Other Network Architectures

**Figure 6:**
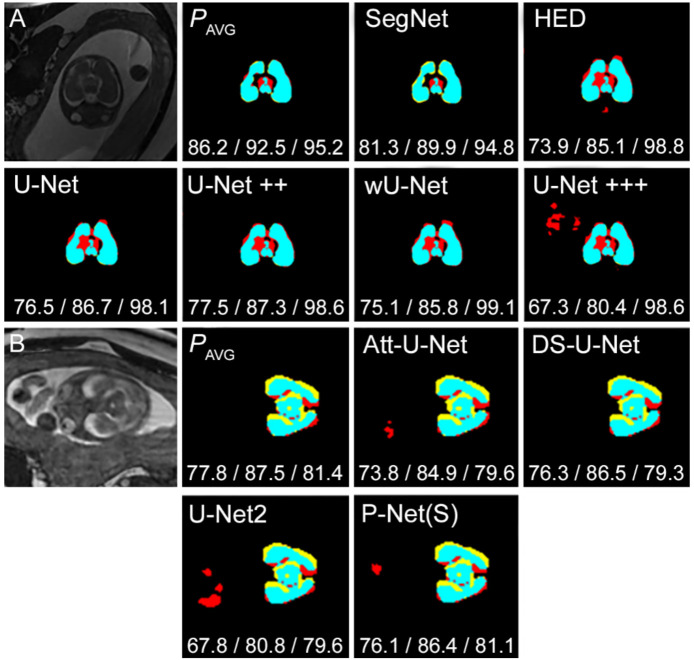
Two segmentation examples (**A**,**B**) providing a visual comparison between SIMOU-Net (*P*_AVG_) and (**A**) SegNet, HED, the original U-Net and its variants and (**B**) some of the latest networks, Att-U-Net, DS-U-Net, U-Net2 and P-Net(S). Black, cyan, red and yellow represent *TN*, *TP*, *FP* and *FN*, respectively. Jaccard/Dice/Sensitivity scores are shown under each segmentation.

Figure 6A provides a visual comparison between the segmentation results produced by the SIMOU-Net and the original U-Net (and its variants), HED [5] and SegNet [31]. In example A, the upper part of the brain is overlapping with the skull. All architectures except the SegNet over segmented this region resulting in false positives (red), whereas SegNet under segmented this region resulting in false negatives (yellow). The SIMOU-Net segments this area with small false positives producing the best results of *J* = 86.2% and *D* = 92.5%. The U-Net+++ (with deep supervision) exhibits overfitting, producing the worst results of *J* = 67.3% and *D* = 80.4%.

In Figure 6B, we compare the SIMOU-Net with some of the latest network architectures specifically designed for foetal brain segmentation. Our method outperformed the U-Net2 [12] by at least 8% for *J* and *D* metrics. However, it only produced marginally better results across different metrics, particularly when compared to the DS-U-Net and P-Net(S).

### 4.5. Segmentation of Challenging Cases

**Figure 7:**
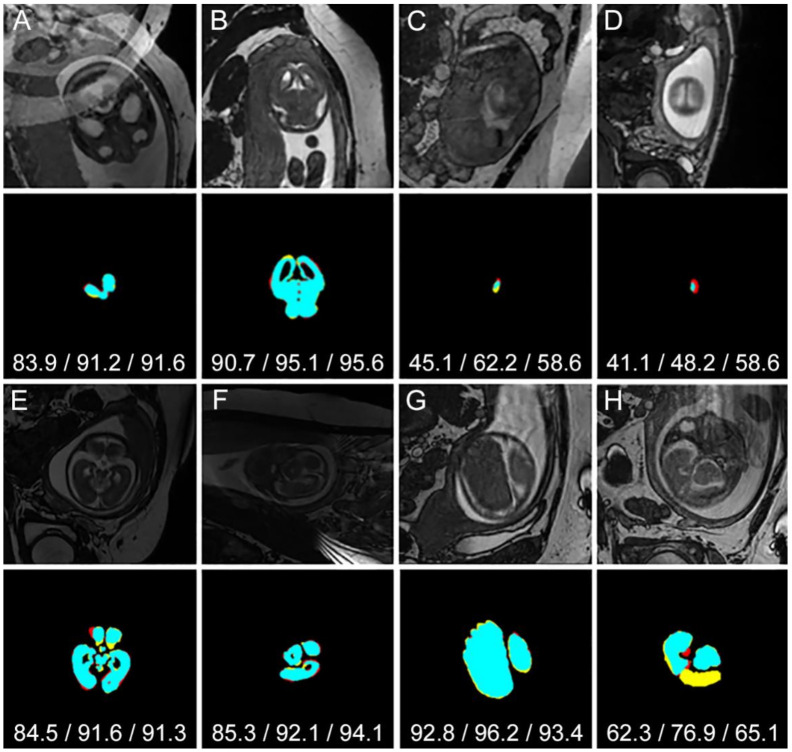
Examples of *P*_AVG_ segmentation results for challenging cases (**A**–**D**) and for abnormal cases; mild ventriculomegaly (**E**), agenesis of corpus callosum (**F**) and structurally deformed (**G**,**H**). Black, cyan, red and yellow represent *TN*, *TP*, *FP* and *FN*, respectively. Jaccard/Dice/Sensitivity scores are shown under each segmentation.

Figure 7 shows example images and quantitative results for some of the challenging cases. Example A shows the brain region overlapping with a ghosting artefact and the proposed method still achieved a credible *D* = 91.2%. In example B, the boundary of the brain is obscured as it is overlapping with the skull. In this case, the proposed method achieved *D* = 95.1%. In more challenging cases (examples C and D), the region is very small and obscured making it very difficult to detect even for experienced observers. In examples C and D, the SIMOU-Net produced Dice scores of 62.2% and 48.2%, respectively. The bottom half of Figure 7 shows quantitative results for abnormal cases (agenesis of corpus callosum (ACC), mild ventriculomegaly (MV) and structurally deformed). Example E shows an MV case where SIMOU-Net produced *D* = 91.6%. For the ACC case in example F, the image suffers from low contrast, but the proposed method still managed to produce *D* = 92.1%. Structurally deformed cases can be seen in examples G and H.

### 4.6. Ensemble Learning on the Original U-Net and Att-U-Net

We conducted another experiment by employing the SIMO approach (taking into account features from side outputs) on the original U-Net and Att-U-Net. Table 3 indicates that ensemble learning increased the overall results across different metrics for both backbone architectures. For example, the original U-Net (*P*_{*f*}_) produced *J* = 79.2%, *D* = 86.8% and *Sen* = 92.4%, but fusing different probability maps using the ‘average’ operation (*P*_AVG_) achieved *J* = 83.2% (+4%), *D* = 89.2% (+2.9%), *Sen* = 90.1% (+1.3%). When using the ‘median’ operation, the ensemble learning approach improved the results by *J* = +3.5%, *D* = +1.7%, *Sen* = +0.4% compared to the original U-Net. The original Att-U-Net (*P*_{*f*}_) produced *J* = 83.5%, *D* = 90.9% and *Sen* = 95.1%, but fusing different probability maps using the ‘average’ operation (*P*_AVG_) achieved *J* = 85.6% (+2.1%), *D* = 92.5% (+1.6%), *Sen* = 95.2% (+0.1%). However, these differences did not generally reach significance with the exception of the U-Net evaluation base on the *J* metric.

### 4.7. Inter-Observer Evaluation

To further evaluate whether the automated approach is performing at a sufficient level, or not, we compared the automated segmentations to inter-observer measurements. For this purpose, we asked a second observer (who was trained in foetal segmentation by the same expert neuroradiologist as for our ‘gold standard’ observer) to annotate 25 randomly selected cases. Subsequently, we separated the 25 cases from our training sample and used the remaining cases to train and validate the network (150 and 25 cases for training and validation, respectively). Dice scores were calculated between the automated segmentation *P*_AVG_ and ‘gold standard’ observers and between the second and ‘gold standard’ observers. Data were checked for normality using a Shapiro–Wilk test and a paired t-test demonstrated that there was no significant difference in Dice scores between the automated segmentation (*D* = 89.88 ± 2.59) and inter-observer (*D* = 88.58 ± 3.96) situations (*p* = 0.15). Similarly, a one-way ANOVA indicated that there was no significant difference in the total brain volumes measured between the automated segmentation, second observer and ‘gold standard’ (*p* = 0.61), suggesting that the automated method performs comparably to the two trained observers. In addition, the intraclass correlation coefficient was calculated between the automated and two observers’ volume measurements and gave a value of 0.953 which is also indicative of an excellent agreement.

### 4.8. Advantages, Disadvantages and Limitations

The main advantage of the proposed network architecture is that it optimises the model by averaging several activation maps, reducing false positives and negatives. Furthermore, this strategy promotes ensemble learning in the network which reduces the variance of predictions and generalization errors. The network can also recover important cues that might disappear due to repetitive convolution operations in the upsampling and downsampling paths. For example, some cues might only be available in the downsampling path (or at the beginning of the upsampling path) of the network and these cues might disappear after many convolutions. In addition, the concept of global and local information in our study is richer because we not only extract this information from the network’s main convolutional outputs but also from side outputs.

The main disadvantage is that our network is more complex due to more convolutional layers and additional feature maps extracted from the side outputs. As a result, it has more parameters and takes a longer time to train (approximately 24 h depending on the number of training samples). However, the network is still considered to be ‘lighter’ compared to U-Net++ and U-Net+++ [28]. The methods of Zhou et al. [29], despite being capable of producing good results, have complex skip connection paths due to the nested strategy which requires maps from downsampling paths to undergo a dense convolution block. The U-Net [4] and SegNet [33] have simpler architectures, but as shown in Table 1 and Table 2, their performance is lower compared to the recent methods developed in 2018 and 2019 such as the Att-U-Net [34] and DS-U-Net [27]. Hence, there is still room for improvement.

As for the HED [5] and wU-Net [29] networks, they are much simpler alternatives and took less than 10 h to train. Nevertheless, their performances are among the worst in this study. The Att-U-Net [34] is more complex than the classical U-Net but is considered ‘lighter’ than our network. The three pathways method proposed by Salehi et al. [22] produced promising results but requires annotations from different views of the brain such as axial, sagittal and coronal. The entire methodology requires training three networks, eventually merging all outputs, which is time consuming and complex. Recent methods that also show excellent results are the P-Net(S) [26] and DS-U-Net [27]. Nevertheless, both methods require several training runs of the same network for localisation, coarse segmentation and fine segmentation purposes.

While SIMOU-Net (*P*_AVG_) was consistently the best performing network in this study, it should be acknowledged that this network has been developed and optimised for this specific study. This may introduce some positive bias towards its performance, even though we aimed to ensure that the other networks were equally well optimised for the task. This may affect the generalizability of the findings when applied to other similar segmentation problems, although we would expect the relative performances to be similar to that found in this study. Furthermore, while the incorporation of information from the side outputs into the final prediction map consistently improved the performance of the segmentation, the differences were small between *P*_AVG_ and *P*_{*f*}_ in most instances and rarely reached statistical significance.

## 5. Conclusions

We proposed SIMOU-Net, a hybrid network architecture inspired by the original U-Net and HED architectures. SIMOU-Net not only takes advantage of the original U-Net features, which extract location and contextual information, but it also takes account of local and global information extracted from the side outputs. As a result, instead of relying on a single prediction map, as in the original U-Net network, SIMOU-Net produces several prediction maps and then applies ‘averaging’ operations on these maps. This approach reduces both the variance of predictions and the generalisation error, which often leads to better results. Furthermore, we have demonstrated that fusing information from multiple side outputs also improves the performance of other network configurations, suggesting that the approach has more general applicability. Experimental results obtained from foetal brain MRI segmentation are promising and indicate that SIMOU-Net is robust and competitive with similar studies in the literature. For future work, we plan to evaluate SIMOU-Net for multi-class segmentation of the foetal brain and for liver segmentation in the foetus via a transfer learning approach.

## Figures and Tables

**Table 1:** Average quantitative results for 200 normal cases. Each numerical value represents the mean value ± standard deviation from stratified three-fold cross-validation. Bold typeface indicates the best performing method for each metric. * indicates a significant difference with respect to the *P*_AVG_ score after performing a Nemenyi test.

Network	*J* (%)	*D* (%)	*Sen* (%)	*MAVD* (cm^3^)	*HD* (mm)
SIMOU-Net					
*P*_{*d,ux*}_	86.9 ± 7.8	93.8 ± 6.2	95.9 ± 6.5 *	4.5 ± 2.9	7.7 ± 3.7
*P*_{*d,u*}_	86.8 ± 6.9	93.6 ± 6.3	95.7 ± 6.5 *	4.5 ± 2.9	7.7 ± 3.7
*P*_{*d*}_	84.3 ± 8.2 *	88.1 ± 7.3 *	92.1 ± 7.9 *	5.1 ± 3.5	7.9 ± 3.9
*P*_{*u*}_	86.9 ±7.8	93.0 ± 6.2	95.9 ± 6.5 *	4.5 ± 2.9	7.8 ± 3.7
*P*_{*f*}_	86.8 ±7.4	93.2 ± 6.2	95.8 ± 6.4 *	4.5 ± 2.9	7.8 ± 3.7
*P*_MAX_	86.1 ± 7.9 *	91.2 ± 6.6 *	95.8 ± 6.2 *	4.7 ± 3.1	8.1 ± 4.1
*P*_MED_	87.2 ± 6.9	93.3 ± 6.1	95.9 ± 6.2 *	4.4 ± 2.9	7.6 ± 3.7
*P*_AVG_	**88.9 ± 6.9**	**94.2 ± 5.9**	**97.5 ± 6.2**	**4.2 ± 2.7**	**7.5 ± 3.6**
U-Net	79.2 ± 8.1 *	86.8 ± 7.4 *	92.4 ± 7.9 *	6.3 ± 5.7	8.6 ± 4.8
wU-Net	74.9 ± 10.6 *	83.5 ± 9.3 *	92.8 ± 7.6 *	7.6 ± 6.4 *	8.9 ± 4.8
U-Net+++	81.3 ± 7.5 *	90.6 ± 6.8 *	95.9 ± 6.4 *	5.9 ± 5.6	8.1 ± 4.2
U-Net++	80.9 ± 7.6 *	89.7 ±6.6 *	95.7 ± 8.3 *	6.1 ± 5.5	8.4 ± 4.3
HED	76.9 ± 10.9 *	85.6 ± 9.9 *	84.7 ± 9.9 *	7.2 ± 6.4 *	10.1 ± 5.6 *
SegNet	81.9 ± 9.2 *	87.9 ± 9.6 *	90.7 ± 7.6 *	5.5 ± 5.5	8.4 ± 4.7
Att-U-Net	83.5 ± 7.8 *	90.9 ± 6.3 *	95.1 ± 6.4 *	5.1 ± 3.5	8.1 ± 4.0
P-Net(S)	85.4 ± 7.9 *	90.1 ± 7.1 *	94.2 ± 8.5 *	5.1 ± 3.5	8.2 ± 4.3
DS-U-Net	86.9 ± 7.8	92.2 ± 6.5	96.1 ± 8.4 *	4.5 ± 2.9	7.6 ± 3.6
U-Net2	80.3 ± 7.9 *	88.1 ± 6.7 *	92.9 ± 9.1 *	6.1 ± 5.5	8.4 ± 4.3

**Table 2:** Average quantitative results (mean ± standard deviation) trained and validated on 200 normal cases and tested on 54 abnormal cases. Bold typeface indicates the best performing method for each metric. * indicates a significant difference with respect to the *P*_AVG_ score after performing a Nemenyi test.

Network	*J* (%)	*D* (%)	*Sen* (%)	*MAVD* (cm^3^)	*HD* (mm)
SIMOU-Net					
*P*_{*d,ux*}_	83.5 ± 6.8	89.2 ± 6.4	92.3 ± 5.9	7.2 ± 12.1	8.7 ± 12.4
*P*_{*d,u*}_	83.6 ± 6.8	89.4 ± 6.4	92.4 ± 5.9	7.2 ± 12.1	8.7 ± 12.4
*P*_{*d*}_	75.1 ± 6.9 *	80.1 ± 6.2 *	85.3 ± 5.5 *	7.9 ± 12.7	8.9 ± 12.6
*P*_{*u*}_	81.1 ± 6.4 *	81.2 ± 6.0 *	88.3 ± 5.3 *	7.5 ± 12.3	8.8 ± 12.5
*P*_{*f*}_	83.4 ± 6.1	89.1 ± 6.4	92.2 ± 5.9	7.2 ± 12.1	8.7 ± 12.4
*P*_MAX_	77.1± 6.3 *	84.2 ± 6.6 *	89.2 ± 6.2	7.7 ± 12.5	8.8 ± 12.6
*P*_MED_	83.7± 6.6	89.3 ± 6.4	92.4 ± 7.1	7.2 ± 12.1	8.7 ± 12.4
*P*_AVG_	**85.7± 6.6**	**91.2 ± 6.8**	**93.2 ± 6.2**	**7.1 ± 12.1**	**8.5 ± 12.4**
U-Net	76.2 ± 8.2 *	82.8 ± 7.2 *	84.4 ± 7.1 *	7.8 ± 12.5	9.0 ± 12.9
wU-Net	71.1 ± 10.1 *	79.6 ± 9.3 *	80.8 ± 8.6 *	8.1 ± 12.7	9.3 ± 13.2
U-Net+++	76.3 ± 8.5 *	82.9 ± 7.2 *	84.7 ± 7.2 *	7.7 ± 12.5	8.8 ± 12.6
U-Net++	76.9 ± 8.4 *	83.2 ± 6.9 *	85.7 ± 7.1 *	7.7 ± 12.5	10.1 ± 12.8
HED	70.1 ± 12.7 *	75.2 ± 10.2 *	79.6 ± 9.9 *	8.2 ± 12.9	11.5 ± 13.9
SegNet	78.2 ± 10.6 *	84.4 ± 9.1 *	87.9 ± 9.6	7.7 ± 12.5	8.9 ± 12.7
Att-U-Net	79.5 ± 7.8 *	85.9 ± 7.3	87.1 ± 7.1 *	7.6 ± 12.3	8.8 ± 12.7
P-Net(S)	83.9 ± 6.9	89.7 ± 6.5	91.3 ± 6.5	7.2 ± 12.1	8.8 ± 12.4
DS-U-Net	85.5 ± 6.6	90.9 ± 6.5	92.9 ± 6.4	7.1 ± 12.1	8.5 ± 12.6
U-Net2	78.5 ± 7.9 *	84.1 ± 6.9 *	85.9 ± 7.6 *	7.7 ± 12.5	9.0 ± 12.7

**Table 3:** Average quantitative results (mean ± standard deviation) from 200 normal cases employing the SIMO approach to the U-Net and Att-U-Net with three-fold cross-validation. Bold typeface indicates the best performing method for each metric. * indicates a significant difference with respect to the *P*_AVG_ score after performing a Nemenyi test.

Network	*J* (%)	*D* (%)	*Sen* (%)	*MAVD* (cm^3^)	*HD* (mm)
U-Net					
*P*_{*d,ux*}_	80.2 ± 7.9 *	87.2 ± 7.1	92.2 ± 5.3	6.3 ± 5.7	8.6 ± 4.9
*P*_{*d,u*}_	80.5 ± 7.5	88.9 ± 7.1	92.9 ± 5.3	6.2 ± 5.7	8.5 ± 4.7
*P*_{*d*}_	70.2 ± 9.4 *	76.2 ± 9.0 *	79.6 ± 7.9 *	6.7 ± 5.9	9.5 ± 5.2
*P*_{*u*}_	79.0 ± 8.1 *	86.1 ± 7.6 *	91.1 ± 6.6	6.3 ± 5.7	8.6 ± 4.9
*P*_{*f*}_	79.2 ± 8.1 *	86.8 ± 7.4	92.4 ± 6.4	6.3 ± 5.7	8.6 ± 4.8
*P*_MAX_	73.1 ± 9.3 *	79.1 ± 7.9 *	81.1 ± 7.2	6.5 ± 5.8	8.8 ± 5.1
*P*_MED_	82.7 ± 7.6	88.5 ± 6.4	92.8 ± 5.8	6.0 ± 5.7	7.8 ± 4.8
*P*_AVG_	**83.2 ± 7.6**	**89.2 ± 6.2**	**93.7 ± 5.7**	**6.0 ± 5.2**	**7.8 ± 4.5**
Att-U-Net					
*P*_{*d,ux*}_	84.9 ± 6.9	90.4 ± 6.2 *	94.1 ± 5.3	5.6 ± 3.5	8.1 ± 4.2
*P*_{*d,u*}_	84.5 ± 6.8	90.2 ± 6.1 *	93.8 ± 5.4	5.6 ± 3.5	8.1 ± 4.2
*P*_{*d*}_	74.3 ± 8.8 *	79.9 ± 8.7 *	82.6 ± 7.1	6.3 ± 4.2	9.4 ± 5.2
*P*_{*u*}_	81.1 ± 7.9 *	86.8 ± 7.3 *	93.3 ± 6.3	6.1 ± 3.8	9.2 ± 4.9
*P*_{*f*}_	83.5 ± 7.8	90.9 ± 6.3	95.1 ± 6.4	5.5 ± 3.5	8.1 ± 4.0
*P*_MAX_	73.9 ± 9.3 *	80.6 ± 7.6 *	84.2 ± 7.0	6.5 ± 4.1	10.1 ± 6.1
*P*_MED_	84.3 ± 7.2	90.2 ± 6.1 *	94.4 ± 5.3	5.6 ± 3.5	8.3 ± 4.4
*P*_AVG_	85.6 ± 7.2	92.5 ± 6.6	95.2 ± 5.2	4.5 ± 2.9	7.8 ± 4.3

## Data Availability

The data presented in this study are available on request from the corresponding author, subject to ethical considerations.
